# Supplementary material for: PathEX: Make good choice for whole slide image extraction
Source: PLoS One. 2024 Aug 29;19(8):e0304702. doi: 10.1371/journal.pone.0304702 (PMC11361590; doi:10.1371/journal.pone.0304702)
Supplement: S1 Table — (PDF) [file pone.0304702.s001.pdf]

Task	PathEX	Histolab	PyHIST	MONAI	SliDL
Grid tile extraction from holistic WSI	✓	✓	✓	✓	✓
Tile extraction using manual annotation files	✓	×	×	×	✓
Classical tissue filtering (foreground filtering)	✓	✓	×	✓	✓
Ability to backgroud filtering (within annotations)	✓	×	×	×	×
Ability to extract small annotation (not mistakes)	✓	×	×	×	×
Handling of manual multiclass annotations for tile extraction	✓	×	×	✓	✓
Ability to generate manual annotation within the software	×	×	×	✓	×
Ability to resolve small annotation mistakes (loops,overlaps, etc.)	✓	×	×	×	✓
Parses doughnut-hole annotations appropriately	✓	×	×	×	✓
AI-assisted annotation within the software	×	×	×	✓	×
Extracts segmentation masks matching paired with tiles	✓	✓	×	✓	✓
Complex artifact filtering	×	✓	×	✓	✓
Fully automated complex artifact filtering	×	×	×	×	✓
Quality control - automatic detection of slides with unusual colour/texture/artefact properties	×	×	×	×	×
Quality control - automatic detection of possible missing or repeated data	×	×	×	×	×

S1 Table: **Table of comparisons to related methods.**
